# Seizure Susceptibility Prediction in Uncontrolled Epilepsy

**DOI:** 10.3389/fneur.2021.721491

**Published:** 2021-09-13

**Authors:** Nhan Duy Truong, Yikai Yang, Christina Maher, Levin Kuhlmann, Alistair McEwan, Armin Nikpour, Omid Kavehei

**Affiliations:** ^1^Australian Research Council Training Centre for Innovative BioEngineering, School of Biomedical Engineering, Faculty of Engineering, The University of Sydney, Sydney, NSW, Australia; ^2^The University of Sydney Nano Institute, Sydney, NSW, Australia; ^3^Faculty of Information Technology, Monash University, Melbourne, VIC, Australia; ^4^Department of Medicine - St. Vincent's Hospital Melbourne, The University of Melbourne, Fitzroy, VIC, Australia; ^5^Comprehensive Epilepsy Service and Department of Neurology at the Royal Prince Alfred Hospital, Sydney, NSW, Australia; ^6^Faculty of Medicine and Health, Central Clinical School, The University of Sydney, Sydney, NSW, Australia

**Keywords:** epileptic seizure forecasting, probabilistic programming, Bayesian, variational inference, uncertainty level

## Abstract

Epileptic seizure forecasting, combined with the delivery of preventative therapies, holds the potential to greatly improve the quality of life for epilepsy patients and their caregivers. Forecasting seizures could prevent some potentially catastrophic consequences such as injury and death in addition to several potential clinical benefits it may provide for patient care in hospitals. The challenge of seizure forecasting lies within the seemingly unpredictable transitions of brain dynamics into the ictal state. The main body of computational research on determining seizure risk has been focused solely on prediction algorithms, which involves a challenging issue of balancing sensitivity and false alarms. There have been some studies on identifying potential biomarkers for seizure forecasting; however, the questions of “What are the true biomarkers for seizure prediction” or even “Is there a valid biomarker for seizure prediction?” are yet to be fully answered. In this paper, we introduce a tool to facilitate the exploration of the potential biomarkers. We confirm using our tool that interictal slowing activities are a promising biomarker for epileptic seizure susceptibility prediction.

## 1. Introduction

There has been great interest recently in identifying biomarkers for seizure susceptibility by looking into critical transitions in brain dynamics in order to enhance the precision of seizure forecasting in a cohort of patients with focal epilepsy (1–3). These studies often require a very long recording that is not available and, in fact, are critically lacking. Chronic and often intracranial electroencephalogram (EEG) recordings demonstrated some limited evidence of circadian, multidien, and circannual cycles in epileptic brain dynamics (4–6). In determining seizure-risk, we believe that understanding what features or biomarkers in the EEG signals lead to such seizure-risk level.

The availability of a seizure forecasting system that can notify patients or their carers about forthcoming seizure-risk can drastically improve patients' quality of life and the chance to develop innovative interventions and preventative therapies. Many studies have been on forecasting seizures; most of them used the signal-modal approach based on electroencephalogram (EEG) signals. These studies can be grouped into two categories: (1) finding discriminative features with various signal processing and transformation techniques and (2) leveraging deep learning's capability of extracting high-level features. In the first group, the most common approach is to use spatio-temporal correlation features, auto-regressive modeling predictive error, Hjorth parameters, spectral power, energy wavelet coefficients, and other statistics (7, 8). Other discriminative features include phase and amplitude lock values (9), common spatial pattern (10), permutation entropy (11), bispectrum features (12). In the second group, the convolutional neural network (CNN) and recurrent neural network (RNN) have shown their capability to extract high-level features that can be used for forecasting seizures. Particularly, CNN was used on the EEG signal spectrogram (13), raw EEG, and fast Fourier transform (FFT) of raw EEG (14), local mean decomposition of raw EEG (15), and the common spatial pattern of multi-channel EEG signals (16). CNN was also used in unsupervised learning as effective feature extraction for seizure prediction (17). To further extract the temporal characteristics over time-series data, Wei et al. (18) applied CNN with long short-term memory recurrent network on the spectrogram of EEG signals. The combination of convolutional and recurrent neural networks is also effective when using multi-timescale of raw time-series EEG signals (19).

**Figure 1:**
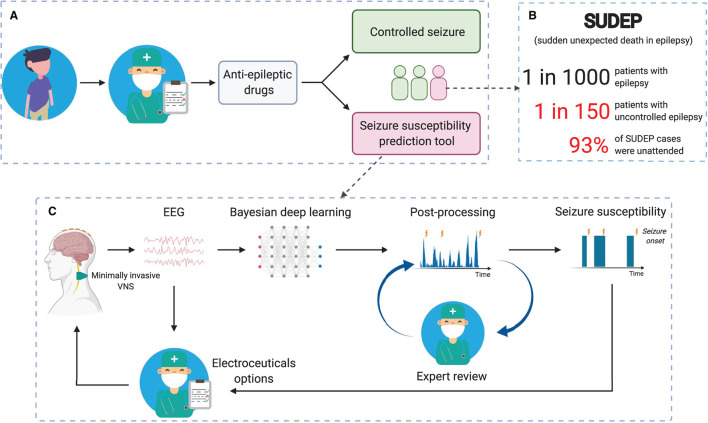
Reducing SUDEP (sudden unexpected death in epilepsy) risk with seizure forecasting based on reliable long-term EEG monitoring. **(A)** Simplified pathway for patients with epilepsy. **(B)** The fact that most SUDEP cases were unattended (20, 21) emerges the need for reliable 24/7 and long-term EEG monitoring. **(C)** Seizure susceptibility prediction tool to suggest alternative treatments [e.g., vagus nerve stimulation (VNS)] for patients with uncontrolled epilepsy.

In this work, we propose a framework (see Figure 1) to minimize the risk of sudden unexpected death in epilepsy (SUDEP), especially for patients with uncontrolled epilepsy. We also introduce a tool to facilitate the exploration of biomarkers for epileptic seizure forecasting. Specifically, we use probabilistic programming and propose a framework to incorporate other relevant information into an EEG-based seizure forecasting system. As an advantage of using probabilistic programming, our system not only can forecast impending seizures but also quantify the uncertainty level of its decision-making.

## 2. Dataset

EPILEPSIAE is the largest epilepsy database that contains EEG data from 275 patients (22). However, up to the time of this writing, only 30 surface EEG and 30 invasive EEG datasets are made available (23). We believe the use of surface EEG is more beneficial because it is non-invasive so it can be applied to a broader group of patients. In this study, we analyze scalp-EEG of 30 patients with 261 leading seizures and 2881.4 interictal hours in total in this work. The time-series EEG signals were recorded at a sampling rate of 256 Hz and from 19 electrodes. Seizure onset information obtained by two methods, namely EEG based and video analysis, is provided. In our study, we use seizure onset information using an EEG based technique, where the onsets were determined by visual inspection of EEG signals performed by an experienced clinician (22). Table 1 provides a summary of the dataset being studied in this work.

**Table 1:** The EPILEPSIAE scalp-EEG dataset.

**Patient**	**Gender**	**Age**	**No. of seizures**	No. of leading seizures^*^	**Interictal hours**
Pat1	Male	36	11	11	68.9
Pat2	Female	46	8	8	114.9
Pat3	Male	41	8	8	96.3
Pat4	Female	67	5	5	126
Pat5	Female	52	8	8	204.1
Pat6	Male	65	8	7	92.2
Pat7	Male	36	5	5	75.7
Pat8	Male	26	22	11	65.6
Pat9	Male	47	6	6	51.1
Pat10	Male	44	11	11	60.7
Pat11	Male	48	14	14	57.8
Pat12	Male	28	9	9	94.1
Pat13	Male	46	8	8	101.3
Pat14	Female	62	6	6	115.7
Pat15	Female	41	5	5	82.8
Pat16	Female	15	6	6	51.1
Pat17	Female	17	9	9	82.4
Pat18	Male	47	7	6	133
Pat19	Male	32	22	21	75.4
Pat20	Male	47	7	7	115.3
Pat21	Female	31	8	8	106.6
Pat22	Male	38	7	7	88.2
Pat23	Male	50	9	9	179.6
Pat24	Female	54	10	10	36.2
Pat25	Male	42	8	8	109.8
Pat26	Male	13	9	9	97.1
Pat27	Male	58	9	8	99.9
Pat28	Female	35	9	9	95.2
Pat29	Male	50	10	10	111.9
Pat30	Female	16	12	12	92.5

* *We are considering leading seizures only. Seizures that are <30 min away from the previous one are considered as one seizure only, and the onset of a leading seizure is used as the onset of the combined seizure*.

## 3. Method

### 3.1. Pre-processing

We split EEG signals into 30-s segments with 50% overlap. We perform a short-time Fourier transform (STFT) with a cosine window of 1-s length and 50% overlap on each 30-s segment and get data with a dimension of (*n* × 59 × 129), where *n* is the number of EEG channels. We remove the first and last two elements along the second axis, which corresponds to time, to eliminate any potential disruption of signal near the signal window's edges. We also remove the dc component of the STFT, which is the first element along the last axis. The final dimension of pre-processed data is (*n* × 56 × 128).

### 3.2. Bayesian Convolutional Neural Network

In this paper, we will use variational inference to approximate posterior densities for Bayesian models (24). Consider *x* = *x*_1 : *n*_ as a set of observed variables and *z* = *z*_1 : *m*_ as a set of hidden variables, with joint density *p*(*z, x*). The inference problem calculates the conditional density of the hidden variables given the observed variables, *p*(*z*|*x*).


(1) $$\begin{array}{l} p\left(z|x\right)=\frac{p\left(z,x\right)}{p\left(x\right)}\;, \end{array}$$

where *p*(*x*) is intractable in many models (24).

Variational inference overcomes this by specifying a variational family Q over the hidden variables (24). The inference problem becomes finding the best candidate q(z)∈Q that is closest in Kullback-Leibler (KL) divergence to *p*(*z*|*x*). The optimization subsequently can be achieved by maximizing a function called the evidence lower bound (ELBO) which is equivalent to minimizing the KL divergence between *q*(*z*) and *p*(*z*|*x*). ELBO is expressed as follows (24):


(2) $$\begin{array}{l} \begin{array}{l} ELBO\left(q\right)=𝔼[\log p\left(z,x\right)]-𝔼[\log q\left(z\right)]\\ \;=𝔼[\log p\left(x|z\right)]+𝔼[\log p\left(z\right)]-𝔼[\log q\left(z\right)]\\ \;=𝔼[\log p\left(x|z\right)]-\text{KL}[q\left(z\right)\|p\left(z\right)] \end{array} \end{array}$$

The stochastic variational inference was proposed by Hoffman et al. (25) to help Bayesian neural networks scale efficiently to large datasets. Particularly, this method generates noisy estimates of the natural gradient of the ELBO by repeatedly sub-sampling (mini-batch) the dataset. The loss function can be defined as the negative of ELBO, i.e., minimizing the loss is equivalent to maximizing the ELBO.


(3) $$\begin{array}{l} loss=-ELBO\left(q\right)=-𝔼[\log p\left(x|z\right)]+\text{KL}(q\left(z\right)\|p\left(z\right)) \end{array}$$

In an EEG-based seizure prediction system, *x* is the EEG signals, and *z* is a variable indicating a seizure to occur in the time window T=[SPH:SPH+SOP]. *SPH* stands for seizure prediction horizon that is defined as the period where seizure should not occur after an alarm rises. *SOP* stands for seizure occurrence period that is defined as the interval where seizure onset is expected to occur (26).

### 3.3. Probabilistic Convolutional Neural Network With Data Fusion

In this section, we will incorporate signals other than EEG signals into the Bayesian CNN. We want to estimate the probability of having a seizure given EEG signals, *p*(*z*|*x*), which is the Bayesian CNN's output. Besides EEG signals, we have other relevant data and want to combine all the seizure forecasting information. Circadian information or time of the day has been used to improve the performance of a seizure prediction system (27). For another instance, electrocardiogram that could change around and even before seizure onsets has been shown helpful in predicting epileptic seizures (28, 29). Other physiological signals that have been observed to change prior to seizure onset, such as blood oxygenation, metabolism, can be used as auxiliary data for seizure prediction (30, 31).

Let us start with EEG signals and one extra signal called *d*. Using Bayes theorem, the posterior probability of having a seizure in the time-window T can be expressed as:


(4) $$\begin{array}{l} p\left(z|x,d\right)=\frac{p\left(d|z,x\right)p\left(z|x\right)}{p\left(d|x\right)} \end{array}$$

Assume *x* and *d* are independent, (e.g., EEG signals are independent with the time of the day and can be considered independent with blood oxygenation), we can rewrite (4) as follows.


(5) $$\begin{array}{l} p\left(z|x,d\right)=\frac{p\left(d|z\right)p\left(z|x\right)}{p\left(d\right)} \end{array}$$

Similarly, for two extra signals, *d*_1_ and *d*_2_ with an assumption that *x*, *d*_1_, and *d*_2_ are independent of each other (e.g., time of the day and blood oxygenation), the posterior probability of having seizure in the time window T can be expressed as:


(6) $$\begin{array}{l} \begin{array}{l} p\left(z|x,d_{1},d_{2}\right)=\frac{p\left(d_{1}|z,d_{2}\right)p\left(z|x,d_{2}\right)}{p\left(d_{1}|d_{2}\right)}\\ \;=\frac{p\left(d_{1}|z\right)p\left(z|x,d_{2}\right)}{p\left(d_{1}\right)} \end{array} \end{array}$$

By substituting Equation (5) (with *d* replaced by *d*_2_) to Equation (6), we have:


(7) $$\begin{array}{l} p\left(z|x,d_{1},d_{2}\right)=\frac{p\left(d_{1}|z\right)p\left(d_{2}|z\right)p\left(z|x\right)}{p\left(d_{1}\right)p\left(d_{2}\right)} \end{array}$$

**Figure 2:**
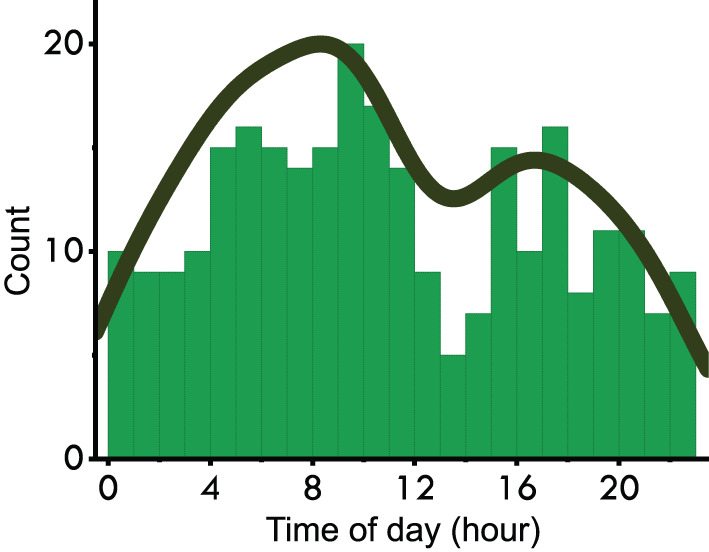
Distribution of time-of-day of seizure occurrences in the EPILEPSIAE scalp-EEG dataset.

To estimate *p*(*d*_1_|*z*) and *p*(*d*_2_|*z*), we applied a kernel density estimation using Gaussian kernels on a histogram containing time of the day (ToD) of seizure occurrences (see Figure 2) (32). Regarding the kernel density estimation parameters, we used Scott's rule for bandwidth selection and assumed all data points are equally weighted. Note that here we approximate $p\left(d_{1}|z\right)\approx p\left(d_{1}|z^{\prime }\right)$ and $p\left(d_{2}|z\right)\approx p\left(d_{2}|z^{\prime }\right)$, where *z*′ is the variable indicating an occurrence of seizure. The approximation is reasonable because we choose the time window T=[5:35 min] which is <1 h.

**Figure 3:**
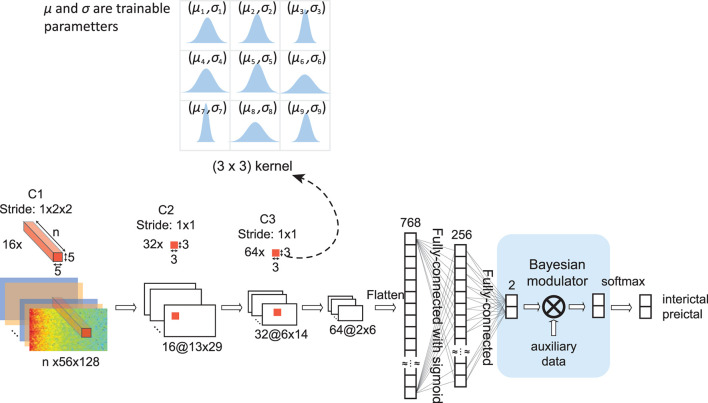
Architecture of Bayesian convolutional neural network (BCNN) with embedded Bayesian modulator. *n* is the number of EEG channels, which is 19 for all 30 patients in this study. Unlike a conventional convolutional neural network where each weight is a single value, each weight of a BCNN is a distribution. In our work, we model each weight as a normal distribution with mean value (μ) and standard deviation (σ) are trainable parameters. The BCNN network has three convolutional layers, each is followed by a max-pooling layer (not shown). Extracted features by the convolutional layers are fed to two fully-connected layers. The Bayesian modulator incorporates other relevant data for seizure forecasting into the last fully-connected layer of the network by using Equation (8).

To incorporate Equation (7) into the training of the Bayesian CNN, we modify the output of the last fully-connected layer (see Fig. 3), before softmax activation (33) as follows.


(8) $$\begin{array}{l} {\text{new-output}}_{l}=\frac{p\left(d_{1}|z^{\prime }\right)p\left(d_{2}|z^{\prime }\right)\times {\text{output}}_{l}}{p\left(d_{1}\right)p\left(d_{2}\right)}, \end{array}$$

where $p\left(d_{1}|z^{\prime }\right)$ and $p\left(d_{2}|z^{\prime }\right)$ can be derived from the kernel density estimation. For example of time of the day, *p*(*d*_1_) = 1/24 because the probability of having the auxiliary signal at a given hour is 1/24); $p\left(d_{1}|z^{\prime }\right)$ can be inferred from Figure 2. Note that Equations (7) and (8) can be extended with more extra signals *d* given that they are independent on each other.

The Bayesian convolutional neural network (BCNN) with Bayesian modulator as data fusion is depicted in Figure 3. Unlike a conventional CNN, where each weight is a single value, each weight of a BCNN is a distribution estimated during the training phase. In this work, we model each weight as a Gaussian distribution with mean and standard deviation values are trainable parameters. Input to the BCNN is the STFT of 30-second windows with size of (*n* × 56 × 128) (see Session 3.1 for details). The network starts with a convolutional layer consisted of 16 3-dimensional kernels of size (*n* × 5 × 5), valid padding, and a stride of (1 × 2 × 2). A max-pooling layer follows the first convolutional layer with a pooling size of (1 × 2 × 2). The network continues with two blocks of convolutional-pooling combinations, each consists of one convolutional layer with a kernel size of (3 × 3), valid padding and stride of (1 × 1), and one max-pooling layer with a pooling size of (2 × 2). The number of convolutional kernels in the two blocks is 64 and 128. The next two layers are fully-connected layers with output sizes of 256 and 2, respectively. The output of the last fully-connected layer is fed to the Bayesian modulator where we apply Equation 8 for data fusion, then is applied softmax activation to get the final output of the network.

## 4. Results

**Figure 4:**
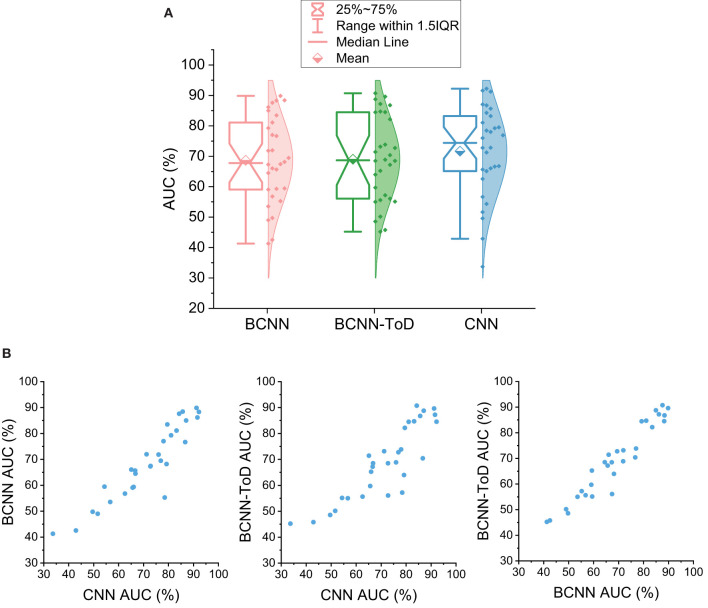
**(A)** Seizure prediction performance using Bayesian convolutional neural network (BCNN). CNN, Convolutional neural network, average AUC is 71.64%; BCNN, BCNN using EEG signals only, average AUC is 68.69%; BCNN-ToD, BCNN using EEG signals and time-of-day (ToD), average AUC is 69.03%. **(B)** Scatter plots between each pair of the three methods showing concordance between them.

This section tests the Bayesian convolutional neural network (BCNN) with the EPILEPSIAE scalp EEG dataset with and without auxiliary signal: time-of-day (ToD). Following Truong et al. (13), we use SPH of 5 min and SOP of 30 for calculating the performance. We also compare a seizure prediction system using a convolutional neural network (CNN) proposed by Truong et al. (13) as a baseline. Figure 4A shows the overall performance of the BCNN with and without auxiliary signal and the baseline CNN. Compared to the CNN that has an average AUC of 71.65%, BCNN achieves an AUC of 68.69% that is around 3% lower than that of CNN. By using the time-of-day information, the overall performance of BCNN-ToD is slightly improved by 0.3–69.03%. There is strong agreement between the methods that is reflected via scatter plots of AUC between each pair of them (see Figure 4B). We did a one-tailed Wilcoxon signed-rank test and a one-tailed *t*-test between BCNN-ToD and CNN and found that the two methods' performance is not significantly different at the confidence level of 0.05 with *p*-values of 0.063 and 0.243, respectively. However, the BCNN-ToD provides more insights into how the prediction works. Particularly, because each weight of the BCNN or BCNN-ToD is a distribution, we sample those distributions to calculate the output for each forward pass of an input. By running multiple forward passes of the same input, we can estimate the distribution of the corresponding output. The output's distribution can then be used to quantify the uncertainty of the model's decision-making that will be explored in section 5.

## 5. Discussion

**Figure 5:**
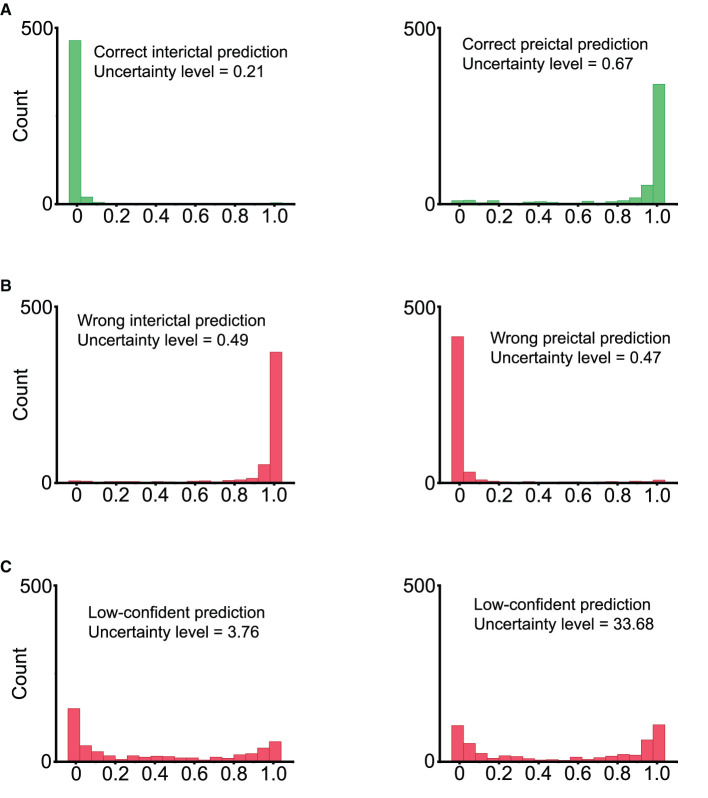
Inference values by sampling the output of Bayesian convolutional neural network 500 times. **(A)** Correct predictions. Left: ground truth is 0 (interictal), most of the model's output samples are close to 0, indicating a correct prediction with high confidence. Right: ground truth is 1 (preictal), most of the model's output samples are close to 1, indicating a correct prediction with high confidence. **(B)** Wrong predictions. Most of the model's output samples are close to the wrong value with high confidence. This is an undesirable case. **(C)** Low-confident predictions with high uncertainty levels. The model's output samples spread randomly between 0 and 1, indicating the high uncertainty of the model.

Bayes convolutional neural network (BCNN) can generate the distribution of its output for each input. We sampled the output of the BCNN by feeding forward the same input through the BCNN 500 times. We quantify the uncertainty level of the BCNN's decision making with Equation (9) below. The numerator takes into account the variability of the output with the standard deviation (std). The denominator considers the case where the output has a uniform-like distribution. Uncertainty levels of different types of prediction distributions are illustrated in Figure 5.


(9) $$\begin{array}{l} \text{Uncertainty level}=\frac{{\text{std}}_{\text{inference values}}}{\left|{\text{mean}}_{\text{inference values}}-0.5\right|} \end{array}$$

**Figure 6:**
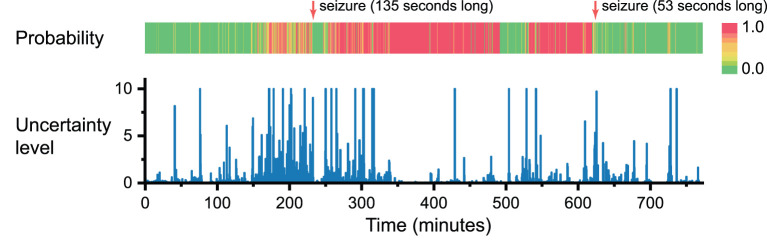
Prediction score and uncertainty level produced by Bayesian convolution neural network in seizure forecasting task over 13 h of continuous EEG recording. For the sake of visualization, for all uncertainty levels higher than 10, we set them to 10.

We trained the BCNN with two types of EEG signals: preictal—35 to 5 min before seizure onset, and interictal—at least 4 h away from any seizures, we are interested in how the BCNN performs with continuous EEG recording. We ran inference over 13 h of continuous EEG recording for one of the best performers, Patient 4, consisting of two seizures. In Figure 6, we plot both the prediction scores (from 0 to 1, where higher values indicate a higher probability of having a seizure) and the corresponding uncertainty levels of the BCNN. In general, the prediction scores get higher values when it is closer to the first seizure onset. Interestingly, at around time 40 and 80 min (around 200 and 160 min before the first seizure onset), there are two predictions with high scores. However, the uncertainty levels were also high, which means that the BCNN “thinks” that there might be a seizure incoming, but it has very low confidence about its decision. From about 1 h before seizure to seizure onset, we can see prediction scores were mostly high, but there were also many low prediction scores with high uncertainty levels. We suggest that the “patterns” or bio-markers related to seizure prediction only occur at certain particular points in time rather than consistently throughout the whole preictal duration.

**Figure 7:**
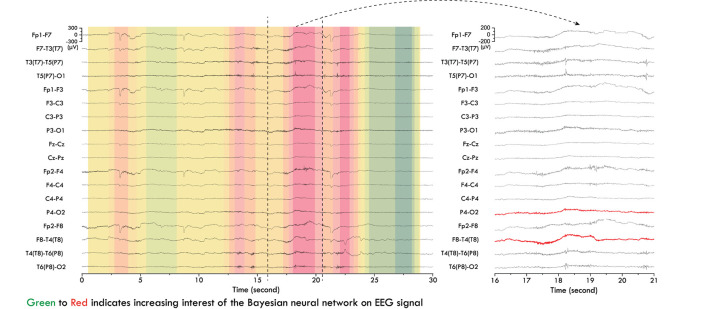
Slow EEG activity as potential bio-marker detected by Bayesian convolutional neural network (BCNN) for epileptic seizure forecasting. Red channels (on the right) were labeled as origin of seizure in the EPILEPSIAE dataset.

Furthermore, we were able to use the trained BCNN as a tool to extract potential bio-markers from EEG signals. We fed 30-s EEG segments to the trained BCNN to sample the output, i.e., run multiple inferences with the same input; in this work, we ran 100 times. Uncertainty level and mean prediction score were extracted from the output samples. For every 30-s segment that has an uncertainty level below 0.1 and means prediction score above 0.9, we extract the attention map over time by accumulating over time the positive values of the feature map of the first convolutional layer. An example is illustrated in Figure 7. We observed that, from patients with high performance and with focal seizures, the BCNN focuses on slow EEG activity when performing seizure forecasting. Slow EEG activity has been shown as an important biomarker for studying epilepsy (34).

**Figure 8:**
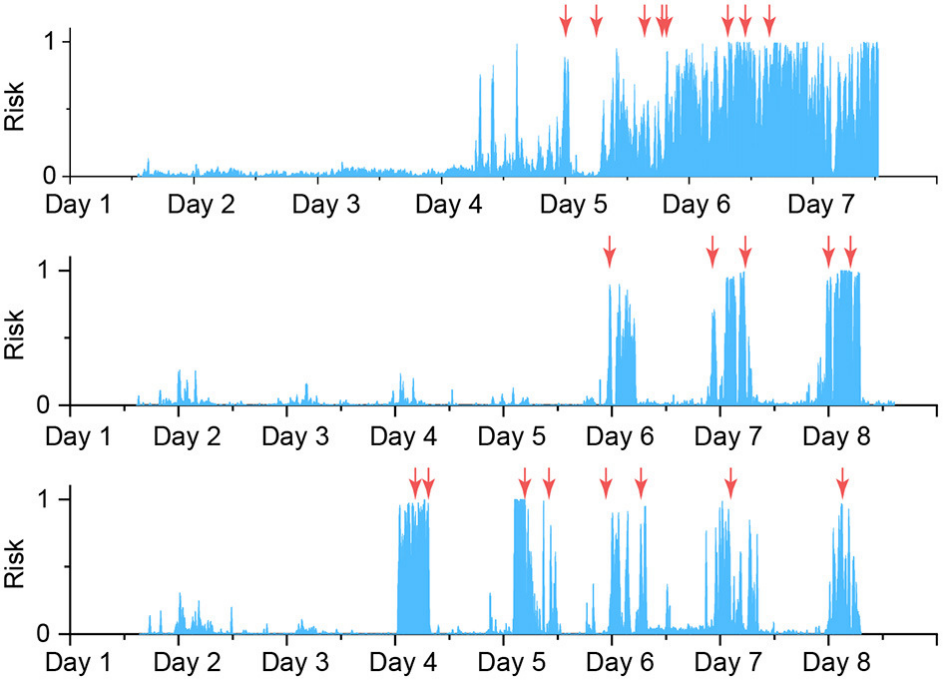
Risk level of having seizures for top three good performance patients, from top to bottom: Pat-3, Pat-4, Pat-12. All decisions with an uncertainty level higher than 1 are discarded and not shown. Moving average of 50 steps backwards is applied to the probability score (risk level). Orange arrows indicate the seizure onsets.

To verify the possibility of seizure forecasting, we ran the inference over the three patients' EEG recordings with the best seizure prediction performance in the EPILEPSIAE dataset, namely Pat-3, Pat-4, and Pat-12. We used different trained BCNN models at different periods separated by ictal segments to ensure that the trained BCNN being used did not see the current period's preictal segment during training. By doing that, we can have a retrospective risk of having a seizure over time, as shown in Figure 8. Generally, the risks are higher when it is closer to the seizure onset, indicating successful seizure forecasting. However, there are cases that we consider as false positive alarms if the risk is assessed as high, but it is too far from the seizure onset, e.g., the day before. For instance, Pat-3 receives a high-risk alarm almost 16 h before the first seizure onset.

Finally, we introduced the Bayesian modulator as a data fusion technique to incorporate relevant auxiliary signals for improving seizure prediction performance. In this work, we have only used one extra signal, which is the time of the day, and the performance was not increased significantly. However, we argue that with the Bayesian modulator's capability to embed multiple auxiliary signals, we can achieve a boost in performance which is our aim in future works. Other relevant signals that can be used for data fusion include heart rate variability, blood oxygenation, metabolism. Some signals that have been shown related to seizure onsets, such as electrodermal activity, near-infrared spectroscopy, skin temperature, and respiratory monitor (35), can be used for data fusion as experimental exploration. For example, to use heart rate variability (HRV) for data fusion, one can plot a distribution of HRV during preictal periods (i.e., 35 to 5 min prior to seizure onset), and then apply the kernel density estimation. Lastly, we relied on the assumption that the auxiliary signals are independent of each other and independent of the main signal, i.e., EEG, to derive Equation 7. We are aware that this assumption may not always be entirely met. However, we argue that machine learning models may still work even if the assumptions are weakly met or violated; e.g., in the field of reinforcement learning, Markov property usually is not satisfied, but many models have shown working effectively in practice (36).

## 6. Conclusion

Epileptic seizure forecasting is still a substantially challenging task, but it has a consequential impact on patients' quality of life and their caregivers. While some patient-specific demonstrated excellent performance in a subset of patients, generalized predictions on non-invasive EEG recordings can work well on most patients, which has been a great challenge. This work presented an innovative approach to incorporate uncertainty and auxiliary signals information in seizure-risk forecasting. These informative warning signals will be invaluable for decision-making in employing any risk-mitigation intervention or therapies. We built our method based on the Bayesian convolutional neural network to provide an insight into the uncertainty level of seizure-risk prediction.

## Code Availability

The code used to generate all results in this manuscript can be made available upon request.

## Data Availability Statement

Publicly available datasets were analyzed in this study. This data can be found at: European database on epilepsy (http://www.epilepsy-database.eu/).

## Author Contributions

NT carried out data analysis and wrote the manuscript. OK suggested the project and supervised the work. AN provided clinical and diagnostic information. YY, CM, LK, AM, and OK revised the manuscript. All authors contributed to the article and approved the submitted version.

## Funding

OK acknowledges the support provided by The University of Sydney through a SOAR Fellowship and Microsoft's support through a Microsoft AI for Accessibility grant.

## Conflict of Interest

The authors declare that the research was conducted in the absence of any commercial or financial relationships that could be construed as a potential conflict of interest.

## Publisher's Note

All claims expressed in this article are solely those of the authors and do not necessarily represent those of their affiliated organizations, or those of the publisher, the editors and the reviewers. Any product that may be evaluated in this article, or claim that may be made by its manufacturer, is not guaranteed or endorsed by the publisher.
